# Gastroprotective Chitosan Nanoparticles Loaded with Oleuropein: An In Vivo Proof of Concept

**DOI:** 10.3390/pharmaceutics16010153

**Published:** 2024-01-22

**Authors:** Hend Abd-Allah, John Youshia, Gehad A. Abdel Jaleel, Azza Hassan, Mevidette El Madani, Maha Nasr

**Affiliations:** 1 Department of Pharmaceutics and Industrial Pharmacy, Faculty of Pharmacy, Ain Shams University, Cairo 11566, Egypt; hend.abdallah@pharma.asu.edu.eg (H.A.-A.);; 2 Pharmacology Department, National Research Centre (NRC), Giza 12622, Egypt; 3 Pathology Department, Faculty of Veterinary Medicine, Cairo University, Cairo 12613, Egypt

**Keywords:** oleuropein, chitosan nanoparticles, gastric ulcer, anti-inflammatory, antioxidant

## Abstract

Oleuropein is the main constituent of olive leaf extract, and it has shown antioxidant and gastroprotective properties against gastric ulcers. Chitosan nanoparticles are known for their mucoadhesive abilities, and consequently, they can increase the retention time of drugs in the gastrointestinal tract. Therefore, loading oleuropein onto chitosan nanoparticles is expected to enhance its biological efficiency. Oleuropein-loaded chitosan nanoparticles were prepared and characterized for particle size, surface charge, in vitro release, and anti-inflammatory activity. Their in vivo efficacy was assessed by measuring specific inflammatory and protective biomarkers, along with histopathological examination. The optimum oleuropein chitosan nanoparticles were cationic, had a size of 174.3 ± 2.4 nm and an entrapment efficiency of 92.81%, and released 70% of oleuropein within 8 h. They recorded a lower IC50 in comparison to oleuropein solutions for membrane stabilization of RBCs (22.6 vs. 25.6 µg/mL) and lipoxygenase inhibition (7.17 vs. 15.6 µg/mL). In an ethanol-induced gastric ulcer in vivo model, they decreased IL-1β, TNF-α, and TBARS levels by 2.1, 1.7, and 1.3 fold, respectively, in comparison to increments caused by exposure to ethanol. Moreover, they increased prostaglandin E2 and catalase enzyme levels by 2.4 and 3.8 fold, respectively. Immunohistochemical examination showed that oleuropein chitosan nanoparticles markedly lowered the expression of IL-6 and caspase-3 in gastric tissues in comparison to oleuropein solution. Overall, oleuropein chitosan nanoparticles showed superior gastroprotective effects to oleuropein solution since comparable effects were demonstrated at a 12-fold lower drug dose, delineating that chitosan nanoparticles indeed enhanced the potency of oleuropein as a gastroprotective agent.

## 1. Introduction

Gastric ulcer is a common disease of the human gastrointestinal tract and has a prevalence of 8.4% [1]. It occurs due to the imbalance between mucosal-defensive factors (gastric mucus, prostaglandins, bicarbonate, and antioxidants) and mucosal offensive factors (gastric HCl, ethanol, free radicals, and *Helicobacter pylori*). Current medical treatment relies on proton pump inhibitors, histamine receptor antagonists, prostaglandin analogs and antibiotics in the case of *Helicobacter pylori* infection. However, they are usually associated with adverse effects and recurrence [2,3]. This has driven the search for natural-based alternatives.

Olive leaf extract is known for its antioxidant activity and was found to have a gastroprotective role against gastric ulcer [4,5,6,7]. This role was attributed to its main constituent oleuropein [2,5]. Oleuropein is a polyphenolic compound that was reported to exert beneficial protective effects against ethanol-induced gastric ulcer in rats, giving the best results at a dose of 12 mg/kg [8]. Additionally, it was found to have similar effects against indomethacin-induced gastric ulcer in rats, though higher doses (250 and 500 mg/kg) were recommended [9].

Chitosan is a cationic linear polysaccharide obtained from chitin, with a degree of deacetylation above 50%. It is formed of linked D-glucosamine and N-acetyl-D-glucosamine units with favorable properties and is safe and biocompatible. Chitosan nanoparticles (CS NPs) have been used extensively in drug delivery, showing promising results for several drugs and various routes of administration [10,11,12]. They have been previously used as a carrier for drugs against ethanol-induced ulcers [3,13,14,15], enhancing the pharmacological response. This was attributed to their cationic charge adhering to the negatively charged mucin lining the gastric mucosa [3]. This mucoadhesion resulted in an increased retention time of nanoparticles in the stomach, achieving higher local drug concentration [13,14]. Moreover, chitosan nanoparticles were chosen as a carrier for oleuropein since the latter was shown to exhibit an affinity to chitosan [16]. In addition, chitosan nanoparticles were reported to efficiently load both hydrophilic and hydrophobic drugs of sufficient water solubility, and hence, oleuropein with a reported aqueous solubility of about 1.7 mg/mL and a log P of −0.865 [17] was a suitable candidate for encapsulation in chitosan nanoparticles.

In this study, our goal was to utilize CS NPs as a drug delivery carrier for oleuropein to enhance its gastroprotective effect. We compared the performance of pretreating rats with oleuropein-loaded CS NPs with that of oleuropein solution against ethanol-induced ulcers. The formulated oleuropein CS NPs were characterized by measuring the particle size, surface charge, and oleuropein release from nanoparticles and by morphological examination under an electron microscope, in addition to assessing the anti-inflammatory activity in vitro. In vivo, we measured specific serum inflammatory markers and local gastroprotective and oxidative stress markers in addition to macroscopic and microscopic examination of the gastric tissue.

## 2. Materials and Methods

### 2.1. Materials

Oleuropein was purchased from the Skinactives Company, Gilbert, AZ, USA. Chitosan (Chitoclear^®^fg 95ULV, molecular weight 77 kDa, degree of deacetylation >95%) was obtained as a gift from the Primex Company, Siglufjordur, Iceland. Sodium tripolyphosphate (TPP), phosphotungstic acid, and glacial acetic acid were purchased from Sigma Aldrich, Gillingham, UK. All the other reagents used were of analytical grade.

### 2.2. Preparation of Oleuropein-Loaded Chitosan Nanoparticles

Oleuropein-loaded chitosan/TPP nanoparticles were prepared using the ionotropic gelation technique [18] applying optimized parameters to produce nanoparticles with a suitable particle size for oral delivery [10]. A volume of 2 mL TPP (0.125% *w*/*v*) was added to 5 mL chitosan solution (0.1% *w*/*v* in 0.1% *v*/*v* acetic acid pH = 5), containing different amounts of oleuropein (3, 5, and 10 mg), in a dropwise manner (drop size 0.05 mL) under magnetic stirring at 25 °C for 30 min (IKA C-MAG HS7, Königswinter, Germany) at a speed of 1000 rpm. The nanoparticles were stored at 2–8 °C for further characterization.

### 2.3. Characterization of Oleuropein Chitosan Nanoparticles

#### 2.3.1. Determination of the Particle Size, Polydispersity Index (PDI), and Zeta Potential (ZP)

The particle size (PS), polydispersity index (PDI), and zeta potential (ZP) of the prepared nanoparticles were determined using a Zetasizer Nano ZS (Malvern, UK) after dilution 100 folds using deionized water and equilibration for one min at 25 °C [10].

#### 2.3.2. Determination of Entrapment Efficiency and Loading of Oleuropein in Chitosan Nanoparticles

Selected chitosan nanoparticles encapsulating oleuropein were centrifuged at 15,000× *g* rpm, 4 °C for 30 min (Hermle Labortechnik GmbH, Model Z216 MK, Wehingen, Germany). The free drug in the supernatant of the selected formulation was determined using a UV spectrophotometer (Shimadzu, Kyoto, Japan) at 280 nm [19]. The entrapment efficiency (EE%) was calculated using the following equations:

$$EE\%=\frac{W_{t}-W_{f}}{W_{t}}\times 100$$

where *W_t_* is the total weight of oleuropein used and *W_f_* is the weight of free oleuropein.

#### 2.3.3. In Vitro Release of Oleuropein from Chitosan Nanoparticles

The in vitro release of oleuropein from CS NPs was determined using the membrane diffusion technique. Briefly, 1 mL of the nanoparticle formulation was placed in the dialysis bag (Dialysis membrane 12,000–14,000 Mwt cut off, Sigma Aldrich, UK) and was placed in vials containing 25 mL 0.1 M HCL with 1% polysorbate 80 to achieve sink conditions. The vials were placed in a shaking water bath (Kottermann, Hanigsen, Germany) shaken at 50 rpm at a temperature of 37 °C ± 0.5. One milliliter of the solution was taken at different time intervals and replenished with the same volume of the dissolution medium. The withdrawn samples were analyzed for the concentration of the drug using UV spectrophotometry at 280 nm [19]. The release data were kinetically analyzed using different kinetic models to determine the mechanism of drug release from the selected formulation by using linear regression analysis in order to find the best fit of the release data to equations representing zero, first, and Higuchi diffusion release models.

#### 2.3.4. Transmission Electron Microscopy (TEM) Imaging of the Selected Chitosan Nanoparticles

The morphology of the selected chitosan nanoparticles was determined after placement on carbon-coated 400-mesh copper grids, followed by removal of the excess liquid and negative staining using 2% phosphotungstic acid (JEOL JEM 1400, Tokyo, Japan).

#### 2.3.5. In Vitro Membrane Stabilization and Anti-Inflammatory Activity

The anti-inflammatory activity of oleuropein solution and oleuropein CS NPs was verified and compared using the membrane stabilization assay [20] and the lipoxygenase inhibition assay [21].

The membrane stabilization potential was tested on human red blood cells (RBCs). They were obtained through the centrifugation of blood at 3000× *g* rpm in heparin-containing tubes, followed by saline washing. Afterward, they were suspended in an isotonic saline solution and placed in well plates, and then, a volume of 100 µL of either oleuropein solution or oleuropein CS NPs at different concentrations was placed in wells as a hypotonic solution [22]. The mixture was left for 10 min, then centrifuged for 3 min at 1300× *g*. Afterward, the optical density was determined at 560 nm using a microplate reader (BioTek, Szada, Hungary). The inhibition percent of RBC hemolysis was calculated as previously reported [20].

Regarding the lipoxygenase inhibition assay, 200 µL of different concentrations of either oleuropein solution or the selected oleuropein CS NPs were analyzed and the lipoxygenase inhibition activity was determined as described elsewhere [21]. Briefly, the tested samples were separately mixed with soybean lipoxygenase solution in a borate buffer with pH 9, followed by incubation with linoleic acid solution. The increase in absorbance at 234 nm was recorded using a microplate reader (BioTek, Szada, Hungary) to calculate the inhibitory activity percentage.

For both assays, the IC_50_ values corresponding to the inhibitory concentration of the samples required to protect 50% of RBCs against hemolysis or reduce the enzyme’s activity by 50% were calculated from the obtained data.

### 2.4. In Vivo Study

#### 2.4.1. Animals

Male albino rats (130–150 g) were obtained from the National Research Centre (Giza, Egypt). The experiment was conducted in compliance with the ARRIVE guidelines [23] after obtaining approval from the research ethics committee of the National Research Centre (Ethical approval number 20174).

#### 2.4.2. Experimental Setup

Twenty rats were divided into four groups, each containing five rats. Group I received oral physiological saline only (control group). Groups II–IV were the ethanol-treated groups. Group II was pretreated with physiological saline, while group III was pretreated with 1 mg/kg oleuropein CS NPs. Finally, group IV was pretreated with 12 mg/kg oleuropein solution, which was the dose that gave best results against ethanol-induced gastric ulcer [8]. All treatments were orally administered daily for 7 days, and then, the rats were deprived of food but allowed free access to water for 24 h. Gastric ulcers were induced by administering absolute ethanol (5 mL/kg) using an oral catheter [24] to all groups except for group I. After 4 h, anesthesia was induced in rats using pentobarbital sodium (35 mg/kg, i.p.), and the blood was collected from the abdominal aorta. The blood was centrifuged at 4000× *g* rpm for 15 min to obtain the serum. The serum was stored at −80 °C for further biochemical assays represented by measuring the inflammatory markers IL-1β and TNF-α (Elabscience Biotechnology Co., Beijing, China). After collection of the blood samples, the rats were sacrificed through cervical decapitation. Their stomachs were excised and rinsed immediately in ice-cold normal saline followed by visual inspection and measurement of the length and number of gastric lesions [25]. Then, the stomach tissue was homogenized, and the following markers were assessed: prostaglandin E2 (PGE2) and nitric oxide (NO) using commercial kits (Elabscience Biotechnology Co., Beijing, China). Additionally, the level of glutathione (GSH) and the activity of the catalase (CAT) enzyme were analyzed as described elsewhere [26,27,28]. Moreover, the level of lipid peroxidation in the stomach tissue was evaluated by measuring the thiobarbituric acid reactive substances (TBARS) following a previously reported method [29] (Elabscience Biotechnology Co., Beijing, China).

The collected stomach tissues were prepared for histological examination and staining using hematoxylin and eosin, and they were assessed for gastric mucosal damage as described previously [30], in which the occurrence of epithelial loss was given a score of 0–3, bleeding was given a score of 0–4, inflammatory cell infiltration was given a score of 0–2, and the total pathologic score was the sum of these three partial scores. In addition, the expression of IL-6 and caspase-3 in the gastric glandular tissues was carried out according to the immunohistochemical procedures previously reported [31].

### 2.5. Statistical Analysis

GraphPad Prism version 7.04 for Windows (GraphPad Software, La Jolla California, San Diego, CA, USA) was used to carry out all the statistical tests. Comparisons between means of different groups were analyzed using one-way analysis of variance (ANOVA) followed by Tukey’s post hoc test. The level of significance was taken as *p* ≤ 0.05.

## 3. Results

### 3.1. Preparation and Characterization of Oleuropein-Loaded Chitosan Nanoparticles

**Figure 1:**
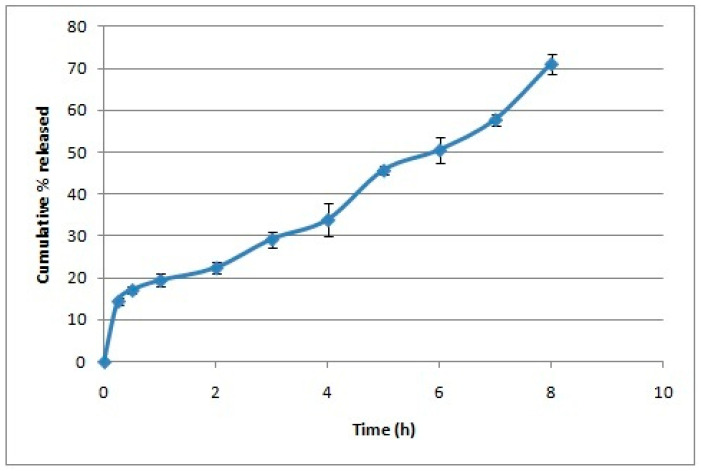
In vitro release of oleuropein from the chitosan nanoparticles over a period of 8 h (*n* = 3).

**Figure 2:**
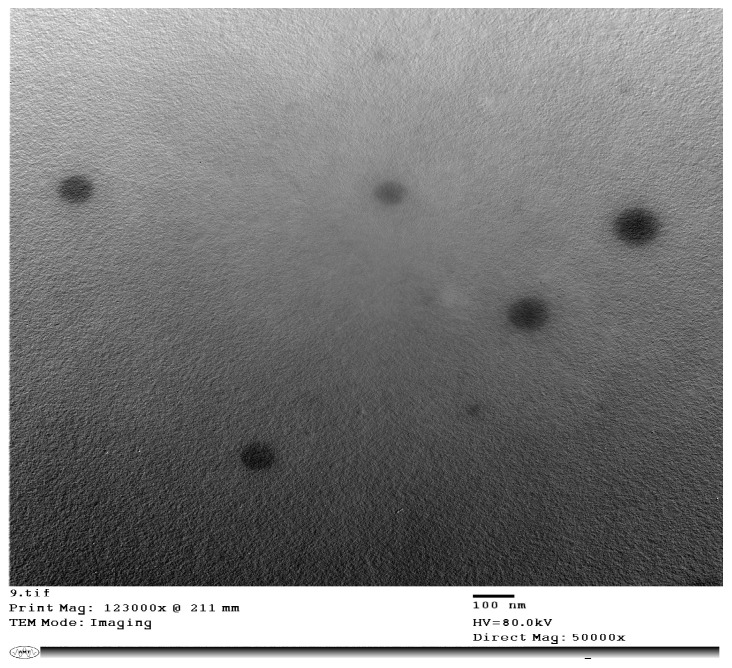
TEM image of oleuropein-loaded chitosan nanoparticles at 50,000× magnification.

Oleuropein-loaded CS NPs were successfully prepared using the ionic gelation technique producing cationic nanoparticles with a PS below 250 nm (Table 1). Adding oleuropein at a concentration of 10 mg/7.5 mg solids resulted in drug precipitation. The one-way ANOVA showed a significant difference among CS NPs formulations in PS, PDI, and ZP. The Tukey post hoc test demonstrated that increasing the amount of oleuropein was accompanied by a significant increase in PS and PDI and a significant decrease in ZP in comparison to plain CS NPs. CS NPs formulated with 3 mg oleuropein showed a smaller PS than those formulated with 5 mg oleuropein, and this was the optimized formula selected for further studies, displaying an EE% of 92.81% ± 8.05. The in vitro release study showed that CS NPs displayed a biphasic pattern in which 20% of oleuropein was released rapidly during the first hour, followed by a more sustained oleuropein release reaching 70% after 8 h (Figure 1), overall following zero-order release kinetics upon fitting to different models. TEM imaging displayed the spherical nature of the chitosan nanoparticles (Figure 2).

### 3.2. In Vitro Anti-Inflammatory Activity

Oleuropein as a solution or entrapped in CS NPs showed anti-inflammatory activity as demonstrated by stabilization of RBC membranes and the inhibition of lipoxygenase enzyme activity (Table 2). Oleuropein CS NPs showed lower IC_50_ for both assays as compared to oleuropein solution.

### 3.3. In Vivo Study

#### 3.3.1. Effect on Serum Inflammatory Markers

Ethanol significantly elevated the serum levels of pro-inflammatory cytokines IL-1β by 3.3 fold and TNF-α by 2.2 fold as compared to the control group (Table 3). Interestingly, pretreating group III rats with oleuropein CS NPs and group IV rats with oleuropein solution significantly reduced the levels of IL-1β and TNF-α as compared to those in group II. Oleuropein CS NPs reduced IL-1β and TNF-α levels by 2.1 and 1.7 fold, respectively in comparison to group II, while the oleuropein solution decreased them by 2.4 and 1.8 fold, respectively. No significant difference was found between oleuropein CS NPs and oleuropein solution, although the former was administered at a 12-fold lower dose than the latter.

#### 3.3.2. Effect on Ethanol-Induced Gastric Lesions

**Figure 3:**
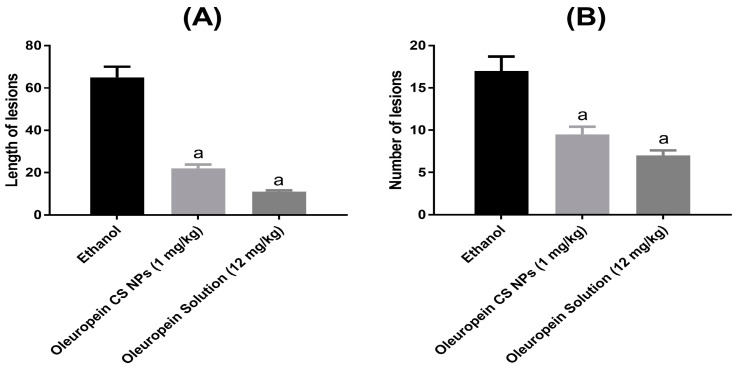
Effect of oleuropein solution and oleuropein CS NPs on (**A**) length and (**B**) number of gastric lesions. ^a^ denotes significant difference from the ethanol group.

The oral administration of ethanol resulted in long and hemorrhagic gastric ulcers in group II rats as demonstrated by the length and number of gastric lesions (Figure 3). However, pretreatment with oleuropein CS NPs and oleuropein solution mitigated the damage caused by ethanol and significantly decreased the number as well as the length of gastric lesions. Similar to serum inflammatory marker levels, no significant difference was found between the oleuropein CS NPs and oleuropein solution groups although the former was administered at a 12-fold lower dose than the latter.

#### 3.3.3. Levels of Gastric Prostaglandin E2 and Nitric Oxide Levels

Results showed that orally administered ethanol significantly reduced gastric PGE2 (4-fold change) and NO (2-fold change) levels in rats. Group III and IV rats pretreated with CS NPs loaded with oleuropein and oleuropein solution showed significantly higher levels of PGE2 in comparison to group II by 2.4 and 2.7 fold, respectively. However, no significant difference was found between both pretreatments and group II in NO levels (Table 3).

#### 3.3.4. Extent of Lipid Peroxidation and Antioxidant Enzymes

As summarized in Table 3, group II rats displayed a significant decrease in GSH (1.9-fold change) and CAT (8.8-fold change) with a significant elevation in TBARS (1.6-fold change) as compared to the normal group. Pretreatment with oleuropein CS NPs and solution significantly depressed the level of TBARS by 1.3 fold, for both, as compared to ethanol. Interestingly, only oleuropein solution could significantly increase GSH levels against ethanol (1.4 fold). As for the CAT enzyme, both pretreatments were successful in significantly increasing its activity as compared to group II (3.8 fold for oleuropein CS NPs and 5.1 fold for oleuropein solution). Group IV rats pretreated with oleuropein solution showed significantly higher CAT activity than group III rats pretreated with oleuropein CS NPs.

#### 3.3.5. Histology and Immunohistochemistry

**Figure 4:**
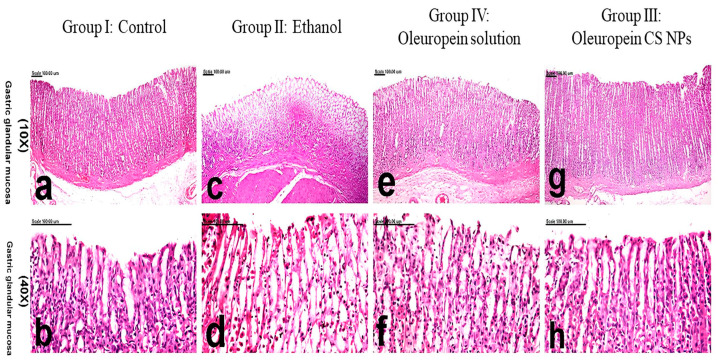
Histological examination of gastric tissue of different animal groups. Group I: control represented by (**a**,**b**), group II: ethanol-induced ulcer pretreated with physiological saline represented by (**c**,**d**), group III: ethanol-induced ulcer pretreated with oleuropein chitosan nanoparticles (CS NPs) represented by (**g**,**h**) and group IV: ethanol-induced ulcer pretreated with oleuropein solution represented by (**e**,**f**).

Examination of the stomach of control rats in group I showed normal histological features with a normal glandular structure and surface epithelium. Additionally, the lamina propria was filled with many tubular gastric glands (Figure 4a,b) and overall scored the lowest pathologic score (Table 4). On the contrary, group II rats suffering from ethanol-induced ulcer displayed complete necrosis and sloughing of the mucosal epithelium extending to the muscularis mucosa (Figure 4c,d), with a significant increase in the pathologic score (Table 4). Hemorrhage and infiltration of leukocytes was detected in the ulcerative lesions. Group IV pretreated with oleuropein solution demonstrated marked improvements, in which the mucosal necrosis was focal, small, and confined to the superficial epithelium. The edema, hemorrhage, and inflammation subsided significantly (Figure 4e,f). In comparison to group II, they recorded a significantly lower pathologic score (Table 4). The most remarkable improvement was demonstrated in group III pretreated with oleuropein CS NPs, in which restoration of the glandular mucosa was evident. Minimal inflammatory reaction and hemorrhage were demonstrated in this group (Figure 4g,h). This group’s pathologic score showed a non-significant difference from the normal group (Table 4).

**Figure 5:**
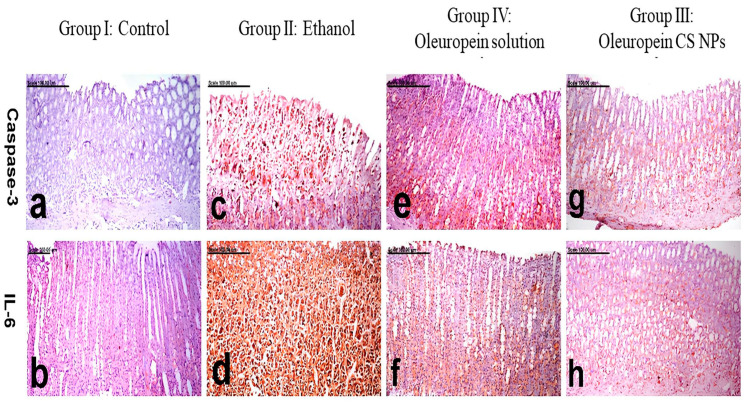
Photomicrograph of gastric mucosa, immunohistochemically stained with IL-6 and caspase-3.Group I: control represented by (**a**,**b**), group II: ethanol-induced ulcer pretreated with physiological saline represented by (**c**,**d**), group III: ethanol-induced ulcer pretreated with oleuropein chitosan nanoparticles (CS NPs) represented by (**g**,**h**), and group IV: ethanol-induced ulcer pretreated with oleuropein solution represented by (**e**,**f**).

The gastric tissues of the normal rats of group I displayed no caspase-3 positively stained cells (Figure 5a), and sparse IL-6 weakly stained cells (Figure 5b). On the other hand, enhanced caspase-3 and IL-6 expressions, with a significant elevation in the percentage of positively stained cells, were recorded in the gastric tissues of group II rats. Caspase-3 and IL-6 immune reactive cells revealed strong brown cytoplasmic and/or nuclear staining (Figure 5c,d). A significant decrease in caspase-3 and IL-6 expressions and a decreased percentage of positively stained cells were recorded in group IV pretreated with oleuropein solution (Figure 5e,f). Minimal expression with a significant decrease in caspase-3 and IL-6 positively stained cells was shown in group III pretreated with oleuropein CS NPs (Figure 5g,h). The oleuropein CS NPs group showed a significantly lower expression of IL-6 and caspase-3 compared to the oleuropein solution group.

## 4. Discussion

In this study, we evaluated CS NPs as drug delivery carriers to augment the protective role of oleuropein against ethanol-induced gastric ulcer in rats. CS NPs were prepared using the ionic gelation method as an easy and reliable method. The optimized formula was cationic owing to chitosan’s amino groups. The produced nanoparticles were spherical and showed a suitable PS of 174.3 nm and a high EE% of 92.81%. A biphasic release pattern for oleuropein was observed, which is typical for CS NPs when releasing drugs in vitro. The in vitro release experiment was carried out in acidic conditions simulating the gastric environment leading to more significant interactions between the protonated amine groups of CS and the water molecules, causing polymer matrix swelling and initial rapid release of the surface-adsorbed drug in the first hour [32]. This is followed by a slower more controlled release of the drug from the core of the nanoparticles through the pores created in the polymer matrix [33]. Furthermore, chitosan nanoparticles loading nicotinamide prepared previously by our research group using the ionic gelation method demonstrated a bioadhesive nature [10], concurring with the mucoadhesiveness of the chitosan polymer, and hence, it was hypothesized that oleuropein chitosan nanoparticles would exhibit desirable mucoadhesive properties, which would positively affect the in vivo study.

The CS NPs maintained the anti-inflammatory activity in vitro as demonstrated in the RBC membrane stabilization and lipoxygenase inhibition assays. The in vivo efficacy was assessed by measuring pro-inflammatory and gastroprotective markers in the blood and gastric homogenate in addition to macroscopic and microscopic examination of the gastric tissue. The serum inflammatory markers included IL-1β and TNF-α, while markers measured in the gastric homogenate included PGE2 and NO representing gastroprotective mediators and GSH, CAT, and TBARS representing oxidative stress indicators. Immunohistochemistry was used to assess the levels of caspase-3 and IL-6 expression in gastric tissues. Although oleuropein CS NPs were administered at a dose 12-fold lower than the oleuropein solution, it showed similar gastroprotective results, and both could mitigate ethanol’s damage.

Ethanol-induced gastric ulcer develops in a relatively short time and therefore resembles acute gastric ulcer in humans; hence, it is the most commonly used in vivo model [34]. Ethanol decreases gastric mucus and induces the formation of reactive oxygen species (ROS). It can dissolve the mucus layer lining the gastric mucosa, increasing its vulnerability to the acidic environment of the stomach. Moreover, it is catalyzed by the alcohol dehydrogenase enzyme to acetaldehyde, which is then metabolized by the xanthine oxidase enzyme producing free radicals and ROS [34]. These ROS increase lipid peroxidation in gastric mucosal cells, affecting their integrity and further damaging the gastric tissue. This damage results in hemorrhage and the formation of lesions. Simultaneously, this initiates an inflammatory reaction, increasing blood levels of IL-1β and TNF-α, which are potent pro-inflammatory cytokine proteins secreted by innate immune cells. Pretreatment of rats with oleuropein CS NPs and oleuropein solution counteracted this inflammatory reaction initiated by ethanol and successfully lowered the serum levels of IL-1β and TNF-α maintaining them close to their normal levels.

Focusing on the local environment of gastric tissue, the oral administration of oleuropein CS NPs and solution showed a macroscopically prominent protective effect as they successfully reduced the length and number of gastric lesions. Regarding the expression of certain biomarkers in the gastric tissues, we measured levels of PGE2 and NO as indicators of mucosal-defensive markers. PGE2 and NO are known to have gastric protective roles against development of gastric ulcers. PGE2 acts by reducing gastric acid production, stimulating mucus secretion, and facilitating healing of gastric ulcers [35]. NO can increase mucosal blood flow and mucus production and limit the infiltration of neutrophils [36,37]. Both markers were decreased on exposure to ethanol but only PGE2 could be raised through pretreatment with oleuropein CS NPs and solution. This could be related to the complex functions associated with the role of NO in gastric ulcers [38].

The oxidative stress was assessed by determining the levels of GSH, CAT, and TBARS. GSH and CAT are critical in protecting cells from ROS. GSH is a major antioxidant that is responsible for the removal of free radicals, while catalase enzyme facilitates the elimination of hydrogen peroxide by decomposing it into water and oxygen. Since ethanol-induced ulcer is usually associated with production of ROS [34], this resulted in the consumption of GSH and CAT, lowering their levels in the gastric tissue. This was accompanied by increased lipid peroxidation as evident by the higher levels of TBARS. Oleuropein is known for its antioxidant activity and ability to scavenge free radicals and ROS. This is attributed to its nature as a polyphenolic compound rich in hydroxyl groups with hydrogen-donating capabilities [39]. Owing to these properties, it was able to reduce the burden of free radicals and ROS following an ethanol insult. This allowed it to restore the levels of GSH in rats pretreated with its solution and CAT activity in rats pretreated with CS NPs entrapping oleuropein and solution as well.

IL-6 is a pro-inflammatory marker, while caspase-3 is a marker for apoptosis. Both increased in ulcerated gastric tissues upon exposure to ethanol. Pretreatment with oleuropein CS NPs and solution lowered the expression of both markers, with oleuropein CS NPs displaying better results. This indicates that both were able to reduce inflammation and avoid excessive death of gastric cells.

Overall, the antioxidant properties of oleuropein were evident in this study. Oral administration of oleuropein as either CS NPs or solution demonstrated gastroprotective results against ethanol-induced ulcer in rats. CS NPs successfully lowered the effective dose of oleuropein. This higher potency of oleuropein CS NPs can be attributed to chitosan’s mucoadhesive properties owing to its cationic nature facilitating interaction with the negatively charged mucin. This was previously reported to increase the retention time of CS NPs in the stomach improving the local drug concentration [3,13,14], and hence, it can be concluded that the combined mucoadhesive and sustained-release properties of oleuropein CS NPs led to prolonged therapeutic action and enhanced drug absorption.

## 5. Conclusions

Oleuropein chitosan nanoparticles displayed favorable physicochemical properties, in terms of the particle size, zeta potential, polydispersity index, and entrapment efficiency of oleuropein. Moreover, they exhibited significant in vitro membrane stabilization and anti-inflammatory activity compared to the unencapsulated oleuropein. Furthermore, oleuropein chitosan nanoparticles showed higher potency than oleuropein solution in ethanol-induced gastric ulcer by manifesting similar gastroprotective effects despite being administered at a dose 12-fold lower than that of the oleuropein solution. This was evident by the significant reduction in IL-1β, TNF-α, and TBARS levels; the elevation of prostaglandin E2 and catalase enzyme levels; and the lowering of the expression of IL-6 and caspase-3 in gastric tissues. Further studies should test oleuropein chitosan nanoparticles against other models of gastric ulcer and other diseases manifested by oxidative stress.

## Figures and Tables

**Table 1:** Characterization of oleuropein-loaded chitosan nanoparticles.

Drug Loading (mg Drug/mg Solids) *	Particle Size (nm)	PDI	Zeta Potential (mV)
-	156.2 ± 3.00	0.16 ± 0.01	+15.2 ± 0.1
3 mg/7.5 mg	174.3 ^a^ ± 2.4	0.33 ^a^ ± 0.016	+11.2 ^a^ ± 0.2
5 mg/7.5 mg	208.6 ^a,b^ ± 3.4	0.38 ^a,b^ ± 0.01	+13.0 ^a,b^ ± 0.5
10 mg/7.5 mg	Drug precipitated		

* All formulations were prepared by adding 2 mL of 0.125% sodium tripolyphosphate to 5 mL of 0.1% chitosan at pH = 5. ^a^ and ^b^ denote a statistically significant difference from the plain and 3 mg/7.5 mg, respectively, at *p* ≤ 0.05 using one-way analysis of variance (ANOVA) followed by Tukey as a post hoc test. Solids refer to the nanoparticulate-forming materials chitosan and TPP.

**Table 2:** Anti-inflammatory activity of oleuropein solution and oleuropein entrapped in chitosan nanoparticles measured through the stabilization of red blood cell membranes and the inhibition of lipoxygenase enzyme.

Membrane Stabilization			Lipoxygenase Inhibitory %		
Sample Concentration (µg/ mL)	Oleuropein Solution	Oleuropein CS NPs	Sample Concentration (µg/ mL)	Oleuropein Solution	Oleuropein CS NPs
1000	89.32 ± 2.3	92.34 ± 0.11	125	81.64 ± 0.34	97.83 ± 1.6
500	80.19 ± 1.5	86.35 ± 3.20	62.5	74.09 ± 0.64	91.15 ± 2.1
250	72.98 ± 1.3	79.38 ± 0.34	31.25	61.91 ± 0.72	86.37 ± 0.92
125	68.91 ± 0.58	71.82 ± 0.92	15.63	50.08 ± 0.89	76.35 ± 1.41
62.5	61.80 ± 1.5	68.24 ± 1.73	7.81	31.25 ± 1.37	55.08 ± 0.58
31.25	56.32 ± 3.1	59.73 ± 2.41	3.9	24.69 ± 2.64	23.13 ± 2.64
15.63	38.94 ± 2.5	42.19 ± 2.03	1.95	15.62 ± 1.7	18.95 ± 1.7
7.81	21.06 ± 1.5	35.76 ± 1.04	0.98	7.2 ± 0.63	8.21 ± 2.1
0	0	0	0	0	0
IC_50_	25.6	22.6	IC50	15.6	7.17

**Table 3:** Levels of serum inflammatory markers, prostaglandin E2, nitric oxide, and antioxidant enzymes for the different groups.

Marker	Group I (Normal)	Group II (Ethanol)	Group III (Oleuropein CS NPs)	Group IV (Oleuropein Solution)
IL-1 beta (pg/mg ptn)	5.37 ± 0.32	17.88 ^a^ ± 1.84	8.64 ^b^ ± 0.51	7.54 ^b^ ± 0.48
TNF alpha (ng/mL)	302.00 ± 20.60	667.86 ^a^ ± 25.50	400.13 ^b^ ± 30.70	377.71 ^b^ ± 18.80
PGE2 (Pg/mL)	3.19 ± 0.18	0.81 ^a^ ± 0.12	1.98 ^a,b^ ± 0.05	2.18 ^a,b^ ± 0.15
NO (uM/mgprot)	48.17 ± 4.30	23.82 ^a^ ± 1.50	27.58 ^a^ ± 0.91	32.71 ^a^ ± 3.1
GSH (umol/g ptn)	1.12 ± 0.05	0.61 ^a^ ± 0.02	0.73 ^a^ ± 0.02	0.85 ^a,b^ ± 0.04
Catalase (U/g.tissue)	363.78 ± 21.50	41.49 ^a^ ± 3.70	157.87 ^a,b^ ± 15.50	264.67 ^a,b,c^ ± 10.40
TBARS (nmol/mg ptn)	92.90 ± 0.97	150.32 ^a^ ± 3.50	119.36 ^a,b^ ± 1.60	115.74 ^a,b^ ± 2.90

Data are represented as mean ± SEM (n = 5), where ^a^, ^b^ and ^c^ denote a statistically significant difference from normal, ethanol and oleuropein chitosan nanoparticles (CS NPs) groups, respectively at *p* ≤ 0.05 using one way analysis of variance (ANOVA) followed by Tukey as a post-hoc test.

**Table 4:** Pathologic scores recorded for the gastric tissues of normal and treated rats.

Group	Pathologic Score
Group I (Control)	0.30 ± 0.21
Group II (Ethanol-induced ulcer pretreated with physiological saline)	5.70 ^a^ ± 0.21
Group III (Ethanol-induced ulcer pretreated with oleuropein CS NPs)	0.40 ^b,d^ ± 0.12
Group IV (Ethanol-induced ulcer pretreated with oleuropein solution)	1.50 ^a,b^ ± 0.34

^a,b,d^ denotes statistically significant difference from group I, group II, and group IV, respectively, at *p* ≤ 0.05 using one-way analysis of variance (ANOVA) followed by Tukey as a post hoc test.

## Data Availability

The datasets for this work are available from the authors upon request.

## References

[1] Salari N., Darvishi N., Shohaimi S., Bartina Y., Ahmadipanah M., Salari H.R., Mohammadi M. (2022). The Global Prevalence of Peptic Ulcer in the World: A Systematic Review and Meta-Analysis. Indian J. Surg..

[2] Sumbul S., Ahmad M.A., Mohd A., Mohd A. (2011). Role of Phenolic Compounds in Peptic Ulcer: An Overview. J. Pharm. Bioallied Sci..

[3] Aman R.M., Zaghloul R.A., El-Dahhan M.S. (2021). Formulation, Optimization and Characterization of Allantoin-Loaded Chitosan Nanoparticles to Alleviate Ethanol-Induced Gastric Ulcer: In-Vitro and in-Vivo Studies. Sci. Rep..

[4] Dekanski D., Ristić S., Mitrović D.M. (2009). Antioxidant Effect of Dry Olive (*Olea europaea* L.) Leaf Extract on Ethanol-Induced Gastric Lesions in Rats. Mediterr. J. Nutr. Metab..

[5] Dragana D., Snežana J.-H., Vanja T., Goran M., Arsić I., Dušan M.M. (2009). Phytochemical Analysis and Gastroprotective Activity of an Olive Leaf Extract. J. Serb. Chem. Soc..

[6] Al-Quraishy S., Othman M.S., Dkhil M.A., Abdel Moneim A.E. (2017). Olive (*Olea europaea*) Leaf Methanolic Extract Prevents HCl/Ethanol-Induced Gastritis in Rats by Attenuating Inflammation and Augmenting Antioxidant Enzyme Activities. Biomed. Pharmacother..

[7] Musa A., Shady N.H., Ahmed S.R., Alnusaire T.S., Sayed A.M., Alowaiesh B.F., Sabouni I., Al-Sanea M.M., Mostafa E.M., Youssif K.A. (2021). Antiulcer Potential of *Olea europea* L. Cv. Arbequina Leaf Extract Supported by Metabolic Profiling and Molecular Docking. Antioxidants.

[8] Alirezaei M., Dezfoulian O., Neamati S., Rashidipour M., Tanideh N., Kheradmand A. (2012). Oleuropein Prevents Ethanol-Induced Gastric Ulcers via Elevation of Antioxidant Enzyme Activities in Rats. J. Physiol. Biochem..

[9] Koc K., Cerig S., Ucar S., Colak S., Bakir M., Erol H.S., Yildirim S., Hosseinigouzdagani M., Simsek Ozek N., Aysin F. (2020). Gastroprotective Effects of Oleuropein and Thymol on Indomethacin-Induced Gastric Ulcer in Sprague-Dawley Rats. Drug Chem. Toxicol..

[10] Abd-Allah H., Abdel-Aziz R.T.A., Nasr M. (2020). Chitosan Nanoparticles Making Their Way to Clinical Practice: A Feasibility Study on Their Topical Use for Acne Treatment. Int. J. Biol. Macromol..

[11] El-Safy S., Tammam S.N., Abdel-Halim M., Ali M.E., Youshia J., Shetab Boushehri M.A., Lamprecht A., Mansour S. (2020). Collagenase Loaded Chitosan Nanoparticles for Digestion of the Collagenous Scar in Liver Fibrosis: The Effect of Chitosan Intrinsic Collagen Binding on the Success of Targeting. Eur. J. Pharm. Biopharm..

[12] Hanna D.M.F., Youshia J., Fahmy S.F., George M.Y. (2023). Nose to Brain Delivery of Naringin-Loaded Chitosan Nanoparticles for Potential Use in Oxaliplatin-Induced Chemobrain in Rats: Impact on Oxidative Stress, cGAS/STING and HMGB1/RAGE/TLR2/MYD88 Inflammatory Axes. Expert Opin. Drug Deliv..

[13] Abd El Hady W.E., Mohamed E.A., Soliman O.A.E.-A., El-Sabbagh H.M. (2019). In Vitro-in Vivo Evaluation of Chitosan-PLGA Nanoparticles for Potentiated Gastric Retention and Anti-Ulcer Activity of Diosmin. Int. J. Nanomed..

[14] Huang Z., Shi Y., Wang H., Chun C., Chen L., Wang K., Lu Z., Zhao Y., Li X. (2021). Protective Effects of Chitosan-Bilirubin Nanoparticles Against Ethanol-Induced Gastric Ulcers. Int. J. Nanomed..

[15] Perumcherry Raman S., Dara P.K., Vijayan D.K., Chatterjee N.S., Raghavankutty M., Mathew S., Ravishankar C.N., Anandan R. (2022). Anti-Ulcerogenic Potential of Anthocyanin-Loaded Chitosan Nanoparticles against Alcohol-HCl Induced Gastric Ulcer in Rats. Nat. Prod. Res..

[16] Katouzian I., Taheri R.A. (2021). Preparation, characterization and release behavior of chitosan-coated nanoliposomes (chitosomes) containing olive leaf extract optimized by response surface methodology. J. Food Sci. Technol..

[17] Sotoudeheian M., Hoseini S., Mirahmadi S.M.S., Farahmandian N., Pazoki-Toroudi H. (2023). Oleuropein as a Therapeutic Agent for Non-alcoholic Fatty Liver Disease During Hepatitis C. Rev. Bras. Farmacogn..

[18] Calvo P., Remuñán-López C., Vila-Jato J.L., Alonso M.J. (1997). Novel Hydrophilic Chitosan-Polyethylene Oxide Nanoparticles as Protein Carriers. J. Appl. Polym. Sci..

[19] El-Gogary R.I., Ragai M.H., Moftah N., Nasr M. (2021). Oleuropein as a novel topical antipsoriatic nutraceutical: Formulation in microemulsion nanocarrier and exploratory clinical appraisal. Expert Opin. Drug Deliv..

[20] Abu-Azzam O., Nasr M. (2020). In Vitro Anti-Inflammatory Potential of Phloretin Microemulsion as a New Formulation for Prospective Treatment of Vaginitis. Pharm. Dev. Technol..

[21] Granica S., Czerwińska M.E., Piwowarski J.P., Ziaja M., Kiss A.K. (2013). Chemical Composition, Antioxidative and Anti-Inflammatory Activity of Extracts Prepared from Aerial Parts of *Oenothera biennis* L. and Oenothera Paradoxa Hudziok Obtained after Seeds Cultivation. J. Agric. Food Chem..

[22] Anosike C.A., Obidoa O., Ezeanyika L.U. (2012). Membrane Stabilization as a Mechanism of the Anti-Inflammatory Activity of Methanol Extract of Garden Egg (*Solanum aethiopicum*). DARU J. Pharm. Sci..

[23] Percie du Sert N., Hurst V., Ahluwalia A., Alam S., Avey M.T., Baker M., Browne W.J., Clark A., Cuthill I.C., Dirnagl U. (2020). The ARRIVE Guidelines 2.0: Updated Guidelines for Reporting Animal Research. PLoS Biol..

[24] Simões S., Lopes R., Campos M.C.D., Marruz M.J., da Cruz M.E.M., Corvo L. (2019). Animal Models of Acute Gastric Mucosal Injury: Macroscopic and Microscopic Evaluation. Anim. Model Exp. Med..

[25] Zhou D., Yang Q., Tian T., Chang Y., Li Y., Duan L.-R., Li H., Wang S.-W. (2020). Gastroprotective Effect of Gallic Acid against Ethanol-Induced Gastric Ulcer in Rats: Involvement of the Nrf2/HO-1 Signaling and Anti-Apoptosis Role. Biomed. Pharmacother..

[26] Góth L. (1991). A Simple Method for Determination of Serum Catalase Activity and Revision of Reference Range. Clin. Chim. Acta.

[27] Habig W.H., Pabst M.J., Jakoby W.B. (1974). Glutathione S-Transferases. The First Enzymatic Step in Mercapturic Acid Formation. J. Biol. Chem..

[28] Singh S., Khajuria A., Taneja S.C., Khajuria R.K., Singh J., Johri R.K., Qazi G.N. (2008). The Gastric Ulcer Protective Effect of Boswellic Acids, a Leukotriene Inhibitor from Boswellia Serrata, in Rats. Phytomedicine.

[29] Ohkawa H., Ohishi N., Yagi K. (1979). Assay for Lipid Peroxides in Animal Tissues by Thiobarbituric Acid Reaction. Anal. Biochem..

[30] Wu X., Huang Q., Xu N., Cai J., Luo D., Zhang Q., Su Z., Gao C., Liu Y. (2018). Antioxidative and Anti-Inflammatory Effects of Water Extract of *Acrostichum aureum* Linn. against Ethanol-Induced Gastric Ulcer in Rats. Evid. Based Complement Altern. Med..

[31] Yahia H., Hassan A., El-Ansary M.R., Al-Shorbagy M.Y., El-Yamany M.F. (2020). IL-6/STAT3 and Adipokine Modulation Using Tocilizumab in Rats with Fructose-Induced Metabolic Syndrome. Naunyn Schmiedebergs Arch. Pharmacol..

[32] Herdiana Y., Wathoni N., Shamsuddin S., Muchtaridi M. (2021). Drug release study of the chitosan-based nanoparticles. Heliyon.

[33] Iacob A.T., Lupascu F.G., Apotrosoaei M., Vasincu I.M., Tauser R.G., Lupascu D., Giusca S.E., Caruntu I.D., Profire L. (2021). Recent Biomedical Approaches for Chitosan Based Materials as Drug Delivery Nanocarriers. Pharmaceutics.

[34] Beiranvand M. (2022). A Review of the Most Common in Vivo Models of Stomach Ulcers and Natural and Synthetic Anti-Ulcer Compounds: A Comparative Systematic Study. Phytomed. Plus.

[35] Takeuchi K., Amagase K. (2018). Roles of Cyclooxygenase, Prostaglandin E2 and EP Receptors in Mucosal Protection and Ulcer Healing in the Gastrointestinal Tract. Curr. Pharm. Des..

[36] Björne H., Petersson J., Phillipson M., Weitzberg E., Holm L., Lundberg J.O. (2004). Nitrite in Saliva Increases Gastric Mucosal Blood Flow and Mucus Thickness. J. Clin. Investig..

[37] Ohta Y., Nishida K. (2002). L-Arginine Protects against Stress-Induced Gastric Mucosal Lesions by Preserving Gastric Mucus. Clin. Exp. Pharmacol. Physiol..

[38] Liang T.-Y., Deng R.-M., Li X., Xu X., Chen G. (2021). The Role of Nitric Oxide in Peptic Ulcer: A Narrative Review. Med. Gas Res..

[39] Hassanzadeh K., Akhtari K., Hassanzadeh H., Zarei S.A., Fakhraei N., Hassanzadeh K. (2014). The Role of Structural C–H Compared with Phenolic OH Sites on the Antioxidant Activity of Oleuropein and Its Derivatives as a Great Non-Flavonoid Family of the Olive Components: A DFT Study. Food Chem..

